# Silica nanoparticles (SiNPs) derived from melon seed husk ameliorate Ni/Al mixture-mediated cognitive impairment in rats

**DOI:** 10.25122/jml-2024-0019

**Published:** 2024-09

**Authors:** Chidinma Promise Anyachor, Chinna Nneka Orish, Anthonet Ndidi Ezejiofor, Ana Cirovic, Aleksandar Cirovic, Baridoo Donatus Dooka, Kenneth Ezealisiji, Orish Ebere Orisakwe

**Affiliations:** 1 African Centre of Excellence for Public Health and Toxicological Research (ACE-PUTOR), University of Port Harcourt, Choba, Port Harcourt, Nigeria; 2 Department of Anatomy, Faculty of Basic Medical Sciences, College of Health Sciences, University of Port Harcourt, Choba, Port Harcourt, Nigeria; 3 Faculty of Medicine, University of Belgrade, Institute of Anatomy, Belgrade, Serbia; 4 Department of Pharmaceutical Chemistry, Faculty of Pharmacy, University of Port Harcourt, Choba, Port Harcourt, Nigeria; 5 Advanced Research Centre, European University of Lefke, Lefke, Northern Cyprus

**Keywords:** aluminum, nickel, Ni/Al mixture, neurotrophic factors, oxido-inflammatory, acetylcholinesterase

## Abstract

This study evaluated the protective effects of silica nanoparticles (SiNPs) derived from melon seed husk ash against the neurotoxic effects of common environmental pollutants, aluminum (Al), nickel (Ni), and their combination in Wistar rats. Ninety-one male Sprague Dawley rats (220–250 g; 6–8 weeks old) were divided into 13 experimental groups. Key findings revealed that exposure to nickel, aluminum, or their combination significantly impaired spatial learning and memory, as evidenced by prolonged latency periods in treated rats. Treatment with SiNPs from melon seed husks reduced these latency periods. Increased Ni and Al levels in the frontal cortex after Ni/Al mixture exposure were mitigated by SiNPs. SiNPs also countered the reduction in iron levels caused by exposure to nickel, aluminum, and the mixture of nickel and aluminum. Moreover, SiNPs ameliorated oxidative stress by reducing MDA levels and increasing antioxidant enzyme activities. SiNPs treatment caused improved nerve growth factor (NGF) and brain-derived neurotrophic factor (BDNF) levels and reversed elevated Aβ-42 and cyclooxygenase-2 levels, highlighting their potential neuroprotective effects. Our results demonstrated the neuroprotective effects of SiNPs from melon seed husks by attenuating metal-induced oxidative stress and inflammation, though they did not enhance cortical cholinergic activity in rats.

## INTRODUCTION

Each year, significant amounts of toxic elements are introduced into the environment from human activities such as mining, smelting, and agricultural practices. Additionally, natural sources, like volcanic eruptions, also contribute metals to the environment [1]. Toxic metals and metalloids are not only found individually but also occur as mixtures in various parts of the ecosystem [2]. Recent attention has been directed towards the presence of these metals in low-dose mixtures [3]. Human exposure to toxic elements primarily occurs through environmental accumulation, with food and water being major exposure pathways [4]. Toxic metals have been linked to various health issues, including hypertension, neurological disorders, cognitive impairments, cerebral palsy, blindness, dysarthria, cancer, cirrhosis, and hyperkeratosis [5-7]. Unlike essential trace elements, which play critical roles in metabolic processes, toxic trace elements are harmful, persistent in the environment, and tend to accumulate through dietary consumption [8]. In contrast, nutritional metals like copper, magnesium, calcium, zinc, and iron are vital for maintaining proper metabolic functions [9].

Aluminum (Al) is a ubiquitous metalloid associated with developing neurodegenerative diseases through multiple signaling pathways. It contributes to the dysfunction and activation of glial cells, which play a key role in maintaining central nervous system homeostasis and neurodevelopment [10,11]. Aluminum exposure leads to oxidative stress, reduced glutathione depletion, mitochondrial function disruptions, and increased production of pro-inflammatory cytokines in the brain [12]. These neurotoxic effects mirror many of the behavioral, neuroanatomical, and neurochemical features of Alzheimer's disease (AD). Specifically, aluminum promotes the production and deposition of amyloid-beta (Aβ) and hyperphosphorylated tau [11] while also causing deficits in cortical cholinergic neurotransmission and inducing ferroptosis [13,14].

Nickel (Ni) is widely used, particularly in stainless steel production [15]. In humans, exposure to Ni primarily occurs through diet, water, air, tobacco smoke, and skin contact with metallic objects such as coins and jewelry [16]. Additional sources include cookware and certain artificial medical implants [17]. Inhalation of nickel carbonyl and nickel bisulfides is associated with severe health effects, including brain damage [15]. Furthermore, plant-based fuels, such as petroleum products, can contribute to both environmental and bodily accumulation of Ni and Al mixtures [18]. Stainless steel cookware has also been identified as a significant source of combined Ni and Al exposure [19-21].

Public health concerns arise from exposure to toxic metals like Ni and Al. One important characteristic of oral adsorbents used in poison management is their ability to strongly bind toxic metals while sparing essential nutritional metals, all with minimal side effects [22]. To date, sodium diethylcarbodithioate has been the primary treatment for nickel poisoning despite limited controlled human trials [22]. Disulfiram (tetraethylthiuram disulfide [TETD]) can also adsorb nickel, but its use is hampered by liver toxicity and the potential redistribution of Ni into the brain [23]. Deferoxamine and deferiprone have been employed for Al poisoning [24,25]. Antidotal therapy with metal chelators remains an effective strategy to reduce the level of toxic elements in the body [25]. However, despite the availability of various chelating agents for treating metal poisoning, there is an ongoing need for safer substances and therapeutic regimens to manage metal(loid) overload.

Neurotrophic factors play a critical role in protecting against neuronal toxicity and promoting peripheral nerve regeneration after chemical injury [25] by affecting neuronal survival and neuronal function [26,27]. Brain-derived neurotrophic factor (BDNF) and nerve growth factor (NGF) are notable members of the neurotrophin family [28]. Alterations in BDNF and NGF levels have been associated with Alzheimer's disease, psychiatric disorders, and other neurodegenerative diseases [29]. Amyloid β-peptide accumulation can lead to oxidative stress, inflammation, and neurotoxicity, which can initiate the pathogenic cascade, ultimately leading to apoptosis and deterioration of the neurotransmission systems [30]. Low BDNF levels have been linked to the pathogenesis of AD.

The brain is particularly vulnerable to damage from oxidative stress [2], a process closely linked to AD [31,32]. Several studies have highlighted the role of environmental factors, including metal exposure, in AD development. These metals accumulate in the brain and modulate amyloid-β formation and deposition [33], contributing to neurooxidative damage [2, 34,35]. Metal imbalance may indeed be a key feature of AD, supporting the potential role of chelation therapy. Chelation of metals such as Al and Ni may offer therapeutic benefits by reducing metal-induced oxidative stress and neurodegeneration.

Although targeting metal disruption in neurodegenerative diseases like AD is a promising strategy [36], classical metal chelators are not currently considered ideal agents for AD treatment [37]. This is despite their potential to prevent metal-mediated Aβ aggregation and reactive oxygen species (ROS) formation [38] due to their non-selective action and interference with essential metals.

Recent research has demonstrated that silica nanoparticles (SiNPs) possess strong adsorption potential. SiNPs are widely used for adsorbing pollutants and have been extensively applied to remove toxins, metal contaminants, dyes, and other unwanted substances from industrial and drinking water, making them relevant for water purification [39]. Their effectiveness in adsorbing azo dyes has been thoroughly investigated and attributed to their large surface area, which enhances adsorption capacity[40]. In this study, melon seed husk, typically considered agro-waste, was evaluated for its ability to mitigate metal(loid)-induced neurotoxicity in small mammals, aiming to valorize this waste product in line with the principles of the circular economy.

Given the limited data on the toxicity of metal(loid) mixtures, this study investigated the mechanisms by which a Ni/Al binary mixture induces frontal cortex injury in rats. The goal was to understand the potential neuronal risks associated with human exposure. We also explored the chelation potency of silica nanoparticles derived from melon seed husk in the rat cerebrum by assessing their effects on oxido-inflammatory and neurotrophic biomarkers.

## Material and Methods

### Preparation of melon seed husk

Melon seed husks were procured from a local market in Nigeria on January 12, 2022, and authenticated by Mr. Ozioko of the Botany Department, University of Nigeria, Nsukka. The husks were washed thoroughly with tap water and dried under direct sunlight for six days. After drying, the husks were packed into heat-resistant Petri dishes, placed in a muffle furnace, and calcinated at 750 °C for six hours. The cooled product yielded off-white ash, which was used for further experimentation. A total of 2.00 kg of melon seed husks were processed to obtain the ash.

### Preparation of SiNPs from melon seed husk ash

The sol-gel precipitation method was adapted to extract silica from the melon seed husk ash. The chemical reactions involved were as follows:

SiO_2_ + 2NaOH → Na_2_SiO_3_ + H_2_O

Na_2_SiO_3_ + H_2_SO_4_ → SiO_2_ + NaSO_4_ + H_2_O

A sample weighing 5.00 g of ash was accurately measured and thoroughly mixed with 0.5 N sodium hydroxide solution in a flask. The resulting solution was heated for 30 minutes in a beaker and allowed to reach room temperature before undergoing filtration. Hydrochloric acid was cautiously added drop by drop until neutralization was achieved. The precipitated silica nanoparticles were washed with deionized water, centrifuged at 5000 rpm for 10 minutes, dried in an oven at 120 °C for 24 hours, and re-calcined at 450 °C for one hour to produce pure silica nanoparticles.

### Percentage yield of silica nanoparticles

The yield of silica nanoparticles from melon seed husk was calculated using the following formula:


$$\begin{array}{lll} \text{Yield} & = & \frac{Mass\;of\;silica\;nanoparticles\;obtained}{Mass\;of\;melon\;seed\;husk\;used}\times 100/1\\ \text{Yield} & = & \frac{200\;g}{2000\;g}\times 100/1\\ \text{Yield} & = & 10\% \end{array}$$

### UV-visible spectroscopy

The formation of SiNPs from melon seed husk ash was observed using a spectrophotometer (Perkin-Elmer Lambda 950 UV/Vis Spectrometer). A portion of the sample was taken before and after calcination of the product obtained in a Quartz cuvette with water as a reference solution and then analyzed for Plasmon absorbance spectra between 200 and 900 nm wavelengths at ambient temperature.

### Experimental groups

Ninety-one male Sprague Dawley rats (220–250 g, 6–8 weeks old) were obtained from the Animal House, University of Port Harcourt, Nigeria. The rats were housed in polypropylene cages under controlled conditions (25 °C, 55% humidity, 12-hour light/dark cycle) and provided ad libitum access to standard chow and water. After a 14-day acclimatization period, the animals were randomly divided into 13 groups of seven rats each, with treatments administered for 90 days according to the protocol in Table 1 [41-43].

**Table 1:** Experimental groups and treatment protocols

Group	Experimental protocol [41-43]
1	Control Deionized Water
2	0.2 mg/kg nickel + 1 mg/kg aluminum
3	0.2 mg/kg nickel
4	1 mg/kg aluminum
5	0.2 mg/kg nickel + 1 mg/kg aluminum + 100 mg/kg SiNPs from melon seed husk extract
6	0.2 mg/kg nickel + 1 mg/kg aluminum + 200 mg/kg SiNPs from melon seed husk extract
7	0.2 mg/kg nickel + 1 mg/kg aluminum + 400 mg/kg SiNPs from melon seed husk extract
8	0.2 mg/kg nickel + 100 mg/kg SiNPs from melon seed husk extract
9	0.2 mg/kg nickel + 200 mg/kg SiNPs from melon seed husk extract
10	1 mg/kg aluminum + 100 mg/kg SiNPs from melon seed husk extract
11	1 mg/kg aluminum + 200 mg/kg SiNPs from melon seed husk extract
12	100 mg/kg SiNPs from melon seed husk extract
13	200 mg/kg SiNPs from melon seed husk extract

Group 1 served as the control group. Groups 2-4 were treated with Al+Ni, Ni, and aluminum, respectively. Groups 5-7 were exposed to Al+Ni and co-treated with SiNPs from melon seed husk extract in doses of 100, 200 and 400 mg/kg, respectively. Groups 8 and 9 received Ni plus 100 and 200 mg/kg of SiNPs from melon seed husk extract, while Groups 10 and 11 were treated with the same doses of seed husk extract using aluminum instead of nickel. Finally, groups 12 and 13 received only 100 mg/kg and 200 mg/kg SiNPs from melon seed husk extract, respectively.

Oral gavage was used to administer heavy metals and SiNPs, with a maximum volume of 1 ml per dose. Aluminum was administered at 1 mg/kg [44] and nickel at 0.2 mg/kg [45] five times a week to simulate occupational exposure. The weight of the rats was measured weekly, while food and fluid intake were recorded daily. The Barnes test was conducted one week prior to the necropsy.

### Behavioral assessment

#### Barnes maze test

The Barnes maze test was utilized to evaluate spatial learning and memory, following the methodology previously outlined in our laboratory [46]. The apparatus for this behavioral test consisted of a 91-cm-diameter gray circular plate positioned 90 cm above the ground, featuring 12 identical holes (3 cm in diameter) evenly spaced around its circumference. Among these holes, one small, dark, recessed chamber, designated as the escape box, was situated underneath, serving as the target location. The maze walls were adorned with various visual cues of distinct shapes and colors. The underlying principle of the Barnes maze behavioral testing relies on the rats' utilization of these visual cues to locate and enter the goal box to escape the aversive environment characterized by bright light.

Rats from different groups, as previously mentioned, underwent testing for five consecutive days, with each day consisting of four trials. At the outset of each trial, the rats were placed within a dark gray starting cylinder, which was lifted after approximately 30 seconds to commence the trial. In cases where the animals failed to enter the correct hole within 300 seconds, they were gently guided into it and then returned to their home cage. During the probe trial, all holes were closed. After 300 seconds, the formerly correct hole was reopened to allow the animals to return to their home cage. Following each trial, the platform was meticulously cleaned using 70% ethanol. Reduced latencies in entering the goal box over successive testing days were employed as an indicator of spatial learning and memory.

### Frontal cortex collection and histological examination

On day 90, rats were anesthetized with intraperitoneal pentobarbitone (rectal temperature maintained at 37 ± 0.5 °C). Animals were placed onto a dissecting board, and the bone overlying the left parietal cortex was removed to harvest the brain. The frontal cortex was excised and divided into two halves: one part was homogenized to give a 10 % (w/v) homogenate in an ice-cold medium containing 50-mM Tris-HCl and 300-mM sucrose (pH 7.4) and was centrifuged at 1800 × g for 10 min at 4 oC. The supernatant (10%) was used for biochemical assays. The other half of each brain was fixed in formalin buffer (10%) for histopathological examination after Toluidine blue staining. The examination was done by a histopathologist blinded to the treatment protocol. The employed procedure assessed the cytoarchitecture in addition to neurodegeneration [30,47]. Undamaged neurons are circular with blue spotted nuclei and clear perinuclear cytoplasm, whereas blighted neurons manifest as impaired nuclei (pyknosis, karyorrhexis, and karyolysis) and cytoplasm with diminution of Toluidine blue affinity.

### Determination of heavy metals

The frontal cortex was dried for 48 hours, weighed, and placed in conical flasks containing 3 ml of HNO^3^ at room temperature until the solution became clear. Then, 1 ml of 30% H^2^O^2^ was added to the samples. Following effervescence, the samples were heated at 80 °C to evaporate the HNO^3^, cooled to room temperature, and the final volume was adjusted to 10 ml with 2% HNO^3^. The samples were then brought to a constant volume. Ni, Al, calcium (Ca), iron (Fe), and magnesium (Mg) were quantified using an Atomic Absorption Spectrometer [48].

### Antioxidant marker analysis

Reduced glutathione (GSH) levels were determined using spectrophotometric quantification of the yellow complex formed by DNTB with sulfhydryl groups at 412 nm [49]. Superoxide dismutase (SOD) activity was assayed following the method of another study [50], which is based on the suppression of pyrogallol auto-oxidation, monitored at 420 nm. The activity of glutathione peroxidase (GPx) was measured following the method of another study [51], based on the reaction of DTNB with the excess glutathione to form a complex that absorbs at 412 nm. Catalase (CAT) activity was determined using the method described by Shangari and O’Brien [52], where ammonium molybdate reacts with hydrogen peroxide (H^2^O^2^) to form a yellow complex, detectable at 405 nm.

### Markers of oxidative stress/damage

Malondialdehyde (MDA) levels were measured as thiobarbituric acid reactive substances (TBARS), following the method described by Buege and Aust [53]. Brain acetylcholinesterase (AChE) levels were quantified colorimetrically using the acetylcholinesterase assay kit from Elabscience, based on the method previously described by Ellman *et al*. [54] to determine cholinergic activity. NGF, BDNF, and cyclooxygenase-2 (COX-2) levels were detected using an ELISA kit (Elabscience), according to the manufacturer's protocol.

### Statistical analysis

Results were shown as mean ± standard deviation (SD). Microsoft Xlstat 2014 was used to perform Analysis of Variance (ANOVA) and Tukey multiple comparison pairwise tests to ascertain if the concentration of the biomarkers was significantly different between groups. Descriptive statistical parameters for biomarker and metal concentrations in the various rat organs were obtained using Pandas. Initially, descriptive statistics were performed on the metal and biomarker concentrations, followed by ANOVA to evaluate significant differences in heavy metal and biomarker concentrations across groups. All differences were considered significant at P < 0.05. Pearson’s R correlation was employed to assess the relationships between biomarkers.

## RESULTS

The UV-visible spectroscopy analysis of silica nanoparticles revealed a plasmon resonance band at 400 nm in a hydrated solution, confirming the formation of silica nanoparticles from melon seed husk ash (Figure S1).

### Characterization of silica nanoparticles

The sol-gel precipitated melon husk (MH) silica nanoparticles were characterized by spectrophotometric and X-ray diffraction (XRD) analysis. Dynamic light scattering (DLS) and zeta size of the fabricated nano-silica were evaluated to identify the average size and stability of the particles using a DLS-Nano 25 model. The average particle size and crystalline nature were also confirmed using XRD (Bruker D8). Further microscopic analysis was conducted using transmission electron microscopy (TEM) to access the shape, size, and discreet nature of the nanoparticles S1. The Image J software was employed in the particle size analysis.

### XRD analysis

The X-ray diffraction (XRD) pattern of the fabricated silica nanoparticles from melon seed husk was recorded at the Department of Chemistry, Rhodes University Nanotechnology Center, Greece, using a Bruker D8 Advanced X-ray diffractometer. The analysis employed CuKα radiation (λ = 1.5406 Ȁ) at 40 kV, with a 2 ⊖/⊖ scanning mode. Data were collected across the 2⊖ range of 10° to 99.998°, in a stepwise proceeding of 0.010° degree. The diffractogram displayed ten prominent peaks at 2⊖ values of 21.103o, 23.204o, 25.501o, 26.202o, 27.305o, 30.208o, 31.308o, 33.802o, 35.502o, and 45.702o corresponding to (022), (024), (026), (026), (028), (111), (112), (120), (128) and (202) integer on the 'hkl' planes respectively. These peaks indicate a discrete, spherical crystalline nature of the silica nanoparticles.

These findings are consistent with data from the International Center for Diffraction Data (ICDD). The Bragg reflection optimum was observed at a 2⊖ value of 20.204⊖, and the full width at half maximum (FWHM) was used to calculate the average particle size of the silica nanoparticles, which was approximately 40 nm, based on the Debye-Scherrer equation:


$$D=\frac{k\lambda }{\beta \cos\theta }$$

where: D = Thickness of the nanocrystal, K = constant,

λ= Braggs angle 2⊖.

Other unassigned crystalline peaks that were not too relevant were also observed in the XRD pattern.

### Dynamic light scattering analysis

The size distribution range of the silica nanoparticles, as determined by dynamic light scattering (DLS), is presented in Figure S2. The nanoparticles had a size distribution ranging from 15 to 45 nm, with a mean particle size of 37 nm. These findings are consistent with the particle size obtained from the X-ray diffraction (XRD) analysis, which indicated an average size of 40 nm (Figure S3). The results were complementary.

### Transmission electron microscopy (TEM) analysis

The ultra-sensitive resolution studies using TEM analysis showed that the nano-silica particles were spherical and discreet, with a size range of approximately 40 nm. However, some particles were monoclinic in appearance (Figure S4).

### Behavioral assessment (Barnes Test)

The Barnes test revealed significantly longer latency periods for rats treated with nickel, aluminum, or the nickel/aluminum mixture when compared to both the control group and rats co-treated with nickel, aluminum, or the nickel/aluminum mixture with silica nanoparticles from melon seed husk. Rats treated with only SiNPs from melon seed husk showed significantly shorter latency periods to locate the escape hole compared to the nickel, aluminum, and nickel/aluminum mixture treated groups, as shown in Table 2. The significant (*P* < 0.05) longer periods taken by animals exposed to Ni, Al, and Ni/Al mixture suggest poor performance in the spatial learning and memory dry-land Barnes test. Interestingly, co-treatment with SiNPs significantly reduced the time (*P* < 0.05).

**Table 2:** Effect of SiNPs from melon seed husk on the Barnes performance test in rats exposed to Ni, Al, and Ni/Al mixture

Group	Time (min)
Deionized H_2_O (control)	0.98 ± 0.32^b^
0.2mg/kg Ni + 1mg/kg Al	3.99 ± 1.16^a^
0.2mg/kg Ni	3.77 ± 1.10^a^
1mg/kg Al	3.21 ± 1.39^a^
0.2mg/kg Ni + 1mg/kg Al + 100mg/kg SiNPs	1.72 ± 0.88^b^
0.2mg/kg Ni + 1mg/kg Al + 200mg/kg SiNPs	1.93 ± 1.09^b^
0.2mg/kg Ni + 1mg/kg Al + 400mg/kg SiNPs	2.27 ± 1.43^ab^
0.2mg/kg Ni + 100mg/kg SiNPs	2.05 ± 1.38^b^
0.2mg/kg Ni + 200mg/kg SiNPs	1.58 ± 0.91^b^
1mg/kg Al + 100mg/kg SiNPs	2.00 ± 1.18^ab^
1mg/kg Al + 200mg/kg SiNPs	1.81 ± 0.99^ab^
100mg/kg SiNPs	1.25 ± 0.59^b^
200mg/kg SiNPs	0.88 ± 0.21^b^

Values = Mean ± SD, *n* = 5. Data with different superscripts (a, b, c) are significantly different from each other (*P* < 0.05); data with the same superscripts are not significantly different. HMM, Heavy Metal Mixture.

### Heavy metal concentrations in the frontal cortex

We found higher levels of nickel and aluminum in the frontal cortex following exposure to nickel/aluminum mixture compared to the control group (*P* < 0.05) (Table 3). Co-treatment of nickel, aluminum, and nickel/aluminum mixture with SiNPs from melon seed husk resulted in a significant (*P* < 0.05) reduction in the levels of Ni and Al in the frontal cortex (Table 3).

**Table 3:** Effect of SiNPs of melon seed husk on Ni and Al concentrations in the frontal cortex of male albino rats exposed to Ni, Al, and Ni/Al mixture

Group	Ni (mg/kg)	Al (mg/kg)
Deionized H_2_O (control)	-----	------
0.2mg/kg Ni + 1mg/kg Al	3.40 0.09^b^	1.13 0.01^c^
0.2mg/kg Ni	2.260.04^b^	-----
1mg/kg Al	-----	1.280.02^c^
0.2mg/kg Ni + 1mg/kg Al + 100mg/kg SiNPs	0.8 ± 0.12^c^	0.15 ± 0.112^a^
0.2mg/kg Ni + 1mg/kg Al + 200mg/kg SiNPs	0.44 ± 0.15^c^	0.05 ± 0.015^b^
0.2mg/kg Ni + 1mg/kg Al + 400mg/kg SiNPs	0.32 ± 0.22^c^	0.11 ± 0.081^a^
0.2mg/kg Ni + 100mg/kg SiNPs	0.41 ± 0.17^c^	------
0.2mg/kg Ni + 200mg/kg SiNPs	0.25 ± 0.15^c^	------
1mg/kg Al + 100mg/kg SiNPs	-------	0.06 ± 0.02^b^
1mg/kg Al + 200mg/kg SiNPs	-------	0.06 ± 0.02^b^
100mg/kg SiNPs		
200mg/kg SiNPs		

Values = Mean ± SD, *n* = 5. Data with different superscripts (a, b, c) are significantly different from each other (*P* < 0.05); data with the same superscripts are not significantly different. HMM, Heavy Metal Mixture.

### Calcium, magnesium, and iron concentrations

The effects of SiNPs from melon seed husk on Ca, Mg, and Fe levels in the frontal cortex of rats exposed to nickel, aluminum, or Ni/Al mixture are shown in Table 4. Compared to controls, aluminum and nickel/aluminum mixture exposure significantly (*P* < 0.05) decreased iron levels in the frontal cortex, whereas Ni exposure did not. Co-treatment with SiNPs significantly (*P* < 0.05) increased Fe levels. Ca levels significantly decreased, and Mg levels increased following nickel, aluminum, and nickel/aluminum mixture treatments. Co-treatment with SiNPs reversed these effects. SiNPs alone reduced the levels of Ca and increased the levels of Mg in both the control and metal-treated groups.

**Table 4:** Effect of SiNPs from melon seed husk on calcium, magnesium, and iron concentrations in the frontal cortex of male albino rats exposed to Ni, Al, and Ni/Al mixture

Groups	Iron (mg/kg)	Calcium (mg/kg)	Magnesium (mg/kg)
Deionized H_2_O (control)	194.81 ± 17.77^c^	313.16 ± 21.43^b^	17.96±6.64^c^
0.2mg/kg Ni + 1mg/kg Al	130.40 ± 09.36^e^	296.16 ± 97.44^b^	37.30±5.98^a^
0.2mg/kg Ni	193.35 ± 72.31^a^	216.00 ± 31.27^a^	35.05±3.73^a^
1mg/kg Al	178.48 ± 57.45^c^	210.94 ± 31.22^b^	29.64±8.33^b^
0.2mg/kg Ni + 1mg/kg Al + 100mg/kg SiNPs	231.09 ± 22.66^d^	595.30 ± 38.74^b^	20.57±8.84^c^
0.2mg/kg Ni + 1mg/kg Al + 200mg/kg SiNPs	392.09 ± 81.65^b^	539.57 ± 32.01^d^	14.21±2.49^d^
0.2mg/kg Ni + 1mg/kg Al + 400mg/kg SiNPs	391.29 ± 28.85^b^	523.96 ± 30.40^c^	19.40±7.68^c^
0.2mg/kg Ni + 100mg/kg SiNPs	353.99 ± 21.91^b^	549.22 ± 31.23^c^	18.22±9.32^c^
0.2mg/kg Ni + 200mg/kg SiNPs	320.33 ± 19.33^b^	538.92 ± 30.44^c^	19.88±1.22^c^
1mg/kg Al + 100mg/kg SiNPs	298.33 ± 18.22^b^	532.33 ± 29.33^c^	20.33±2.88^c^
1mg/kg Al + 200mg/kg SiNPs	302.98 ± 18.22^b^	502.38 ± 34.33^c^	19.93±2.01^c^
100mg/kg SiNPs	237.81 ± 12.38^d^	279.54 ± 26.99^e^	12.00±7.47^c^
200mg/kg SiNPs	324.90 ± 31.47^b^	269.50 ± 14.94^a^	26.97±5.24^b^

Values = Mean ± SD, *n* = 5. Data with different superscripts (a, b, c) are significantly different from each other (*P* < 0.05); data with the same superscripts are not significantly different. HMM, Heavy Metal Mixture.

### Oxidative stress markers

Exposure to nickel, aluminum, and the Ni/Al mixture caused a significant (*P* < 0.05) increase in MDA levels and a decrease in SOD, CAT, GSH, and GPx activities compared to the control group. Co-administration of nickel, aluminum, and nickel/aluminum mixture with SiNPs significantly reduced the levels of MDA and increased SOD, CAT, GSH, and GPx (Table 5; *P* < 0.05). Treatment with SiNPs alone significantly (*P* < 0.05) decreased MDA levels and increased SOD, CAT, GSH, and GPx activities compared to both control and nickel, aluminum, and nickel/aluminum mixture treated groups.

**Table 5:** Effect of SiNPs from melon seed husk on the oxidative stress markers in the frontal cortex of male albino rats exposed to Ni, Al, and Ni/Al mixture

Group	MDA (µmol/ml)	SOD (U/l)	CAT (ug/g)	GSH (ug/ml)	GPx (ug/ml)
Deionized H_2_O (control)	0.24±0.17^b^	0.46±0.40^a^	0.87±0.70^a^	1.78±1.55^a^	0.07±0.01^a^
0.2mg/kg Ni + 1mg/kg Al	0.55±0.21^a^	0.18±0.12^b^	0.38±0.22^c^	0.38±0.02^c^	0.02±0.00^b^
0.2mg/kg Ni	0.55±0.48^a^	0.18±0.12^b^	0.19±0.01^c^	0.39±0.17^c^	0.03±0.01^b^
1mg/kg Al	0.46±0.41^a^	0.17±0.11^b^	0.36±0.17^c^	0.47±0.08^c^	0.03±0.02^b^
0.2mg/kg Ni + 1mg/kg Al + 100mg/kg SiNPs	0.35±0.01^b^	0.24±0.18^c^	0.64±0.48^b^	0.88±0.53^b^	0.09±0.02^a^
0.2mg/kg Ni + 1mg/kg Al + 200mg/kg SiNPs	0.30±0.01^b^	0.19±0.13^b^	1.02±0.86^a^	1.54±1.18^a^	0.06±0.04^ab^
0.2mg/kg Ni + 1mg/kg Al + 400mg/kg SiNPs	0.25±0.01^b^	0.18±0.12^b^	0.83±0.67^a^	1.04±0.68^a^	0.04±0.02^ab^
0.2mg/kg Ni + 100mg/kg SiNPs	0.20±0.13^b^	0.32±0.26^c^	0.75±0.58^a^	0.84±0.62^b^	0.06±0.00^a^
0.2mg/kg Ni + 200mg/kg SiNPs	0.19±0.12^b^	0.23±0.17^c^	0.58±0.41^c^	0.82±0.59^b^	0.07±0.02^a^
1mg/kg Al + 100mg/kg SiNPs	0.17±0.12^c^	0.24±0.18^c^	0.88±0.69^a^	1.64±1.25^a^	0.08±0.01^a^
1mg/kg Al + 200mg/kg SiNPs	0.13±0.08^c^	0.23±0.17^c^	0.74±0.54^ab^	1.64±1.25^a^	0.07±0.02^a^
100mg/kg SiNPs	0.15±0.08^c^	0.23±0.17^c^	1.12±0.95^a^	0.80±0.57^b^	0.05±0.03^a^
200mg/kg SiNPs	0.14±0.07^c^	0.18±0.12^b^	1.26±1.09^a^	0.66±0.44^b^	0.06±0.01^a^

Values = Mean ± SD, *n* = 5. Data with different superscripts (a, b, c) are significantly different from each other (*P* < 0.05); data with the same superscripts are not significantly different. HMM, Heavy Metal Mixture.

### Neurotrophic factors (NGF and BDNF)

Exposure to nickel, aluminum, and nickel/aluminum Ni/Al mixture reduced the levels of NGF and BDNF in the frontal cortex compared to the control group that received deionized water (*P* < 0.05). These effects were significantly reversed by the co-administration of SiNPs. The SiNPs-only treated groups showed significantly higher NGF and BDNF levels than the metal-treated groups (Table 6).

**Table 6:** Effect of SiNPs from melon seed husk on the NGF & BDNF in the frontal cortex of male albino rats exposed to Ni, Al, and Ni/Al mixture

Group	BDNF (pg/ml)	NGF (pg/ml)
Deionized H_2_O (control)	575.00 ± 53.83^a^	858.00 ± 60.34^a^
0.2mg/kg Ni + 1mg/kg Al	285.00 ± 24.95^c^	407.50 ± 43.87^b^
0.2mg/kg Ni	287.50 ± 24.33^c^	436.50 ± 28.84^c^
1mg/kg Al	273.50 ± 21.63^c^	438.00 ± 38.70^c^
0.2mg/kg Ni + 1mg/kg Al + 100mg/kg SiNPs	318.00 ± 27.95^c^	563.50 ± 48.87^b^
0.2mg/kg Ni + 1mg/kg Al + 200mg/kg SiNPs	424.00 ± 38.95^b^	591.00 ± 51.37^b^
0.2mg/kg Ni + 1mg/kg Al + 400mg/kg SiNPs	457.00 ± 41.95^b^	651.50 ± 57.87^b^
0.2mg/kg Ni + 100mg/kg SiNPs	314.00 ± 27.83^b^	561.50 ± 51.84^b^
0.2mg/kg Ni + 200mg/kg SiNPs	490.00 ± 44.83^a^	567.50 ± 51.84^b^
1mg/kg Al + 100mg/kg SiNPs	434.00 ± 37.13^b^	600.00 ± 54.70^b^
1mg/kg Al + 200mg/kg SiNPs	332.00 ± 27.13^bc^	700.50 ± 64.20^b^
100mg/kg SiNPs	372.50 ± 33.33^b^	623.50 ± 57.84^b^
200mg/kg SiNPs	326.00 ± 28.83^b^	639.00 ± 58.34^b^

Values = Mean ± SD, *n* = 5. Data with different superscripts (a, b, c) are significantly different from each other (*P* < 0.05); data with the same superscripts are not significantly different. HMM, Heavy Metal Mixture.

### Aβ-42, AChE, and COX-2 levels

The effects of SiNPs derived from melon seed husk on Aβ-42, AChE, and COX-2 levels in the frontal cortex of rats exposed to nickel, aluminum, and the nickel/aluminum mixture are summarized in Table 7. There was an increase in Aβ-42 and COX-2 levels after treatment with Ni, Al, and Ni/Al mixture compared to the control group. In contrast, AChE activity significantly decreased (*P* < 0.05) following exposure to these metals. These effects were all significantly (*P* < 0.05) reversed by the co-administration of SiNPs (Table 7).

**Table 7:** Effect of SiNPs from melon seed husk on the Aβ-42, AchE, and COX-2 in the frontal cortex of rats exposed to Ni, Al, and Ni/Al mixture

Groups	Aβ-42 (pg/ml)	AChE (ng/ml)	COX-2 (ng/ml)
Deionized H_2_O (control)	19.75 ± 1.75^bc^	260.00 ± 12.23^a^	307.00±24.11^ab^
0.2mg/kg Ni + 1mg/kg Al	33.15 ± 3.81^a^	99.00 ± 18.60^c^	395.00±36.47^a^
0.2mg/kg Ni	27.35 ± 5.35^a^	99.00 ± 30.26^c^	381.50±31.61^a^
1mg/kg Al	31.50 ± 2.14^a^	97.00 ± 17.61^c^	334.50±29.02^a^
0.2mg/kg Ni + 1mg/kg Al + 100mg/kg SiNPs	18.75 ± 1.41^b^	121.50 ± 11.17^b^	291.00±25.47^bc^
0.2mg/kg Ni + 1mg/kg Al + 200mg/kg SiNPs	14.70 ± 2.36^b^	172.50 ± 32.17^b^	280.50±24.97^bc^
0.2mg/kg Ni + 1mg/kg Al + 400mg/kg SiNPs	17.00 ± 1.66^b^	181.00 ± 14.67^b^	312.00±7947^b^
0.2mg/kg Ni + 100mg/kg SiNPs	17.15 ± 1.15^c^	114.00 ± 15.23^b^	316.00±25.11^ab^
0.2mg/kg Ni + 200mg/kg SiNPs	22.55 ± 2.55^b^	106.00 ± 17.23^b^	262.50±20.61^ab^
1mg/kg Al + 100mg/kg SiNPs	17.05 ± 1.69^b^	150.07 ± 17.61^a^	314.50±73.023^ab^
1mg/kg Al + 200mg/kg SiNPs	18.90 ± 1.54^b^	200.08 ± 12.11^a^	313.00±27.52^ab^
100mg/kg SiNPs	17.60 ± 1.60^c^	103.00 ± 14.23^b^	295.50±23.61^ab^
200mg/kg SiNPs	15.80 ± 3.80^c^	134.00 ± 15.23^b^	223.50±16.61^ab^

Values = Mean ± SD, *n* = 5. Data with different superscripts (a, b, c) are significantly different from each other (*P* < 0.05); data with the same superscripts are not significantly different. HMM, Heavy Metal Mixture.

### Correlation between biomarkers

**Figure 1:**
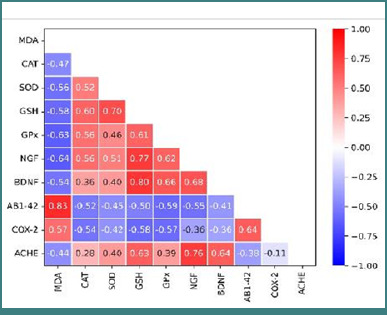
Heat map showing the relationship between the biomarkers and protein in the frontal cortex

The linear relationship between biomarkers in the frontal cortex of rats is presented in Figure 1. AChE and MDA had a negative linear relationship with antioxidant markers. There was a positive relationship between antioxidants, Aβ-42, and COX-2, while antioxidants had a negative correlation with NGF and BDNF. The figure also showed that there was a positive relationship between CAT, SOD, GSH, GPx, and the caspase-3 and BDNF, NGF and AChE. The positive relationship between CAT, SOD, GSH, GPx, NGF, BDNF, and AChE was not significant. A negative relationship was seen between oxidative stress markers and the transcription factors (Aβ-42), which was also significant.

### Histopathological analysis

**Figure 2:**
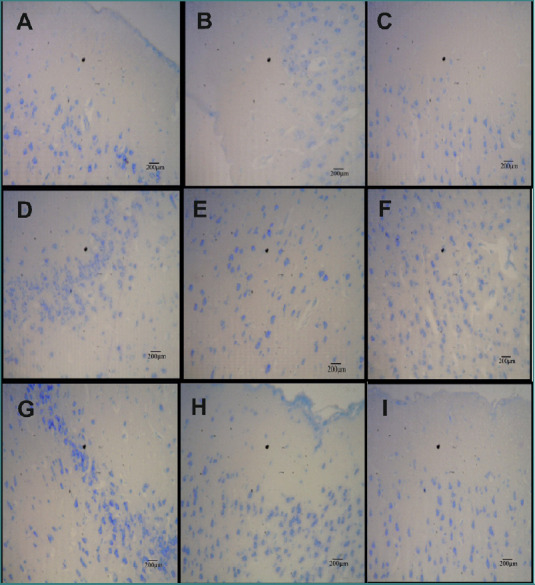
Effects of SiNPs from melon seed husk on the histopathology of the cerebral cortex of male rats exposed to Ni, Al, and Ni/Al binary mixture. A, Toluidine blue-stained cerebral cortex of Group 1 (deionized water) shows normal and intact pyramidal cells at 400X magnification; B, Group 2 (Ni & Al) shows significant neuronal cell loss and vacuolations; C, Group 3 (Ni only) illustrates neuronal cell loss; D, Group 4 (Al only) displays neurodegeneration with vacuolations; E, Group 6 (Ni + Al + 200 mg/kg SiNPs from melon seed husk) shows restoration of neuronal cells; F, Group 7 (Ni + Al + 100 mg/kg SiNPs) indicates increased regeneration of pyramidal cells; G, Group 9 (Ni + 200 mg/kg SiNPs) highlights the regeneration of pyramidal cells; H, Group 12 (100 mg/kg SiNPs) shows intact regenerated pyramidal cells; I, Group 13 (200 mg/kg SiNPs) demonstrates a normal increase in pyramidal cells at 400X magnification.

Histopathological examination of the cerebral cortex in the control group revealed intact histoarchitecture with normal pyramidal cells. In contrast, the groups exposed to the nickel/aluminum binary mixture, nickel alone or aluminum alone, exhibited neuronal cell loss, vacuolations, and neurodegeneration, with vacuolations observed in all treated groups (Figure 2). Co-treatment with increasing doses of SiNPs derived from melon seed husk significantly ameliorated tissue and cellular damage.

Neuronal restoration was observed in Group 6 (Nickel plus aluminum + 200 mg/kg SiNPs), while Group 7 (Nickel plus aluminum + 400 mg/kg SiNPs) demonstrated increased regeneration of pyramidal cells. Group 9 (Nickel + 200 mg/kg SiNPs) also exhibited notable pyramidal cell regeneration. Interestingly, treatment with SiNPs alone at doses of 100 mg/kg and 200 mg/kg (Groups 12 and 13) resulted in intact, regenerated pyramidal cells and normal histoarchitecture (Figure 2).

## Discussion

The possible neuroprotective and neuromodulatory potential of mesoporous silica nanoparticles (SiNPs) derived from melon seed (an agro-waste) was observed in the cerebral cortical histoarchitecture of rats exposed to Ni, Al, and a Ni/Al binary mixture through behavioral and biochemical evaluations. The Barnes performance test was rooted in the understanding that behavior is the net output of memory and cognitive functions within the nervous system and can serve as a sensitive endpoint for chemically induced neurotoxicity [55].

Ni and Al are known neurotoxicants that disrupt the blood-brain barrier (BBB) and cause functional disturbances. These metals can affect ionic and cholinergic neurotransmission, impairing attention, cognitive function, memory, and learning ability [56,57]. The Barnes performance tests demonstrated significantly longer latency in the Ni, Al, Ni/Al binary mixture treated rats.

Under normal physiological conditions, ligands or signaling molecules reaching receptor sites initiate cellular signaling pathways. However, during heavy metal neurotoxicity, these toxicants compete with calcium ions (Ca^2^^+^) at the cell surface, entering through Ca^2^^+^ channels, disrupting the calcium-calmodulin pathway, and preventing signals from reaching the cell nucleus. This interference also leads to the inactivation of the cAMP-response element binding protein (CREB) [58]. The reduction in Ca and Mg levels observed in the Ni, Al, and Ni/Al mixture-treated groups likely contributed to the neurotoxicity and poor performance in the Barnes test. However, co-treatment with SiNPs from melon seed husk restored Ca and Mg levels in the frontal cortex, improving cognitive performance in the affected rats.

The synthesis of cortical proteins, such as BDNF, relies on the activation of CREB, which can be influenced by metal exposure [58]. Immature brain proteins like BDNF, transported via microtubules to presynaptic terminals, require Ca^2^^+^ influx to facilitate their release from vesicles into synapses. However, divalent heavy metals compete with Ca^2^^+^, disrupting this process and hindering the exocytosis of brain proteins, ultimately causing irregularities in neurotransmission and protein secretion [59]. The neuroprotective effects of natural antidotes may arise from their ability to chelate metals or increase intracellular antioxidant levels, thereby regulating cortical protein expression through CREB activation. Heavy metal accumulation in neurons generates ROS and free radicals, contributing to oxidative stress. Natural antidotes can form complexes with metal ions in such cases, reducing ROS and free radical production. This reduction in oxidative stress and free metal ions promotes CREB activation and enhances hippocampal protein expression [58].

Neurotoxicity is triggered by the production of ROS, including superoxide anions, hydrogen peroxide, superoxide radicals, and hydroxyl radicals. The extent of oxidative damage is determined by the balance between oxidative stress and the effectiveness of the body's endogenous antioxidant defense system [60]. The brain is particularly vulnerable to free radical damage due to its high concentration of polyunsaturated fatty acids (PUFAs) [61]. Exposure to metals like nickel and aluminum impairs mitochondrial function and disrupts the antioxidant defense system by reducing antioxidant levels [62]. This study assessed the disruption of lipid metabolism by measuring MDA levels and the activity of key endogenous antioxidant enzymes such as SOD, CAT, GSH, and GPx after exposure to Ni, Al, and the Ni/Al binary mixture, along with co-treatment using SiNPs. Since lipid peroxidation products are one of the primary outcomes associated with oxidative stress, the significantly increased cerebral level of MDA in Ni, Al, Ni/Al binary mixture treated groups in the present study suggests oxidative neurotoxicity [63,64].

SiNPs have been reported to possess antioxidant and anti-inflammatory properties [65], which may explain their ability to reduce lipid peroxidation in this study. The production of superoxide anion and the concomitant activity of its endogenous scavenger-SOD are common features of the pathobiology of many neurodegenerative diseases [60]. Similarly, GPx, which converts lipid peroxides into less harmful alcohols and reduces hydrogen peroxide to water, and CAT, which protects SOD by converting hydrogen peroxide to water and oxygen, are also linked to neurodegenerative conditions like Alzheimer's disease.

In this study, treatment with Ni, Al, and the Ni/Al binary mixture clearly elevated oxidative stress and caused cortical injury, as evidenced by significant reductions in SOD, GPx, and CAT levels compared to the control group. These findings align with previous research showing that metal exposure suppresses antioxidant activity, creating an imbalance between pro-oxidant and antioxidant mechanisms [60,63]. However, co-administration of mesoporous SiNPs derived from melon seed husk effectively counteracted these harmful effects, restoring redox balance within the cerebral cortex of the treated rats.

BDNF and NGF modulation are vital markers for treating or preventing neurologic diseases. There is a profound association between BDNF and NGF and essential metals, which can modulate the neuronal recovery outcome [66]. The neurotrophins BDNF and NGF are associated with the maturation, survival, and maintenance of neuron functions in the central nervous system (CNS) [67], and their decrease gives rise to the development of neuronal injury and neurodegenerative diseases [40]. This study has shown a significant decrease in the brain BDNF and NGF levels after Ni, Al, Ni/Al binary mixture exposure, which agrees with previous findings [68]. The release of these neurotrophins is affected by the displacement of Ca^2^^+^ by Ni and Al, as well as by the generation of ROS, which further impair BDNF and NGF [69,70]. Hence, antioxidants that act on these neurotrophins may be neuroprotective against metal(oids) toxicity. Treatment with SiNPs from melon seed husk or in combination with Ni, Al, Ni/Al binary mixture caused a significant increase in the BDNF and NGF levels compared with the control. The ameliorative effect of SiNPs from melon seed husk on the revamping of BDNF and NGF levels may be ascribed to different processes, including decrement in ROS production, oxidative damage repair, and amplified antioxidative defense mechanisms [71].

BDNF protects neurons against amyloid Aβ peptide toxicity in vivo and in vitro [30], although neurotoxic mechanisms of amyloid peptides remain largely unknown. It is now considered that beta-amyloid peptides may elicit a dysfunctional encoding state in neurons that may trigger neurodegeneration [72] and compromise BDNF signaling during neurodegeneration [73]. The validation of the hypothesis that exogenous BDNF elicits neuroprotection against toxic effects of Aβ peptides in brain regions related to cognitive functions [30] and the observation in this study that SiNPs from melon seed husk reversed the Ni, Al, and Ni/Al mixture-mediated decrease in cortical BDNF levels and increase in Aβ-42 provides a promising new avenue for future therapeutic applications of this previously underutilized agro-waste.

AChE is a proxy biomarker of cortical cholinergic activity in vivo. A decrease in AChE activity, as seen in neurodegenerative diseases like Parkinson's disease (PD), is often linked to impaired cholinergic neurotransmission and cognitive decline [74]. Usually, AChE impedes presynaptic choline uptake/release, which alters neurotransmission, evidenced by substantial neocortical deficits in the enzyme responsible for synthesizing acetylcholine (ACh), choline acetyltransferase (ChAT). There was a significant decrease in AChE in Ni, Al, and Ni/Al mixture-treated groups compared to the control deionized water-treated group. However, co-administration of SiNPs from melon seed husk increased AChE activity, suggesting that while SiNPs may not act directly through cholinergic enhancement, they offer neuroprotection through other mechanisms. This observation agrees with earlier observations [75,76], with the implication that cortical acetylcholine depletion might have no effect on Barnes performance test.

Compared to controls, the reduced AChE levels in Ni, Al, and Ni/Al-treated rats suggest heightened cholinergic activity in these groups. SiNP supplementation increased AChE activity, reversing this effect and indicating that the neuroprotective benefits of SiNPs may not rely on cholinergic enhancement, unlike other natural antidotes like curcumin [77]. There was a significant reduction in AChE in Ni, Al, and Ni/Al combination-exposed groups compared to the control deionized water-treated group in this study. Co-administration of SiNPs from melon seed husk extract with Ni, Al, and Ni/Al combination caused a significant elevation in the AChE compared to Ni, Al, and Ni/Al mixture groups. Acetylcholinesterase AChE metabolizes presynaptic ACh to ensure a rapid and short-lasting action of the cholinergic neurotransmitter.

Some studies have shown that silica nanoparticles possess strong adsorption potential, making them effective in binding and removing pollutants, particularly heavy metals, and emerging metal toxicants [39]. Their large surface area has been identified as the key factor behind their high adsorption capacity, which is central to their mechanism of action [40]. Therefore, the beneficial effects observed in this study could be attributed to the enhanced adsorptive properties of SiNPs.

This study also explored the histological examination of the frontal cortex, revealing the beneficial interventions of SiNPs by mitigating cortical damage caused by exposure to Ni, Al, and their binary mixture. Additionally, the findings highlight the ability of these SiNPs to counteract degenerative changes associated with neuronal disorders. Wolfe and Molinoff [78] suggested that brain degenerative changes could cause Alzheimer's disease in individuals exposed to metal (loids) [78]. This implies the neuroprotective potential of SiNPs from melon seed husks. Perhaps another plausible mode of action of SiNPs from melon seed husk against metal(loids)-induced neurotoxicity might be modulation of cholinesterase activities, which attenuate antioxidant status, anti-inflammatory action, and nerve growth factors associated with neurological diseases.

Although the present study has captured more absorption, distribution, metabolism, and excretion (ADME) considerations than toxicodynamics, further studies will focus on potential adverse outcome pathways (AOPs) to elucidate the mode-of-action of main events that lead to adverse outcomes in the downstream of the key molecular initiating events and subcellular neurotoxicity pathways. It can be suggested that SiNPs derived from melon seed husk may be beneficial in managing metal(loid)-induced neurotoxicity as demonstrated by the improvements observed in behavioral and biochemical markers and confirmed by histological examination. These effects were achieved through upregulating neurotrophic factors BDNF, NGF, enhanced antioxidant, anti-inflammatory properties, and biometal chelation of SiNPs from melon seed husk.

## CONCLUSION

Overall, this study demonstrated that SiNPs from melon seed husk mediated learning and memory impairment in rats induced by nickel, aluminum, and their binary mixture. The behavioral recovery was driven by the augmented cerebral cortex antioxidant and neurotrophic factor levels that lowered the neurotoxic Aβ-42 and boosted noxious biometal chelation with the enhancement of magnesium level in the cerebral cortex.

## Supplementary Material



## Personal thanks

All authors are grateful to anonymous reviewers for the comments provided in improving the manuscript.
