# Supplementary material for: Silica nanoparticles (SiNPs) derived from melon seed husk ameliorate Ni/Al mixture-mediated cognitive impairment in rats
Source: J Med Life. 2024 Sep;17(9):856–67. doi: 10.25122/jml-2024-0019 (PMC11611061; doi:10.25122/jml-2024-0019)
Supplement: Supplementary file 1 [file JMedLife-17-856-s001.pdf]

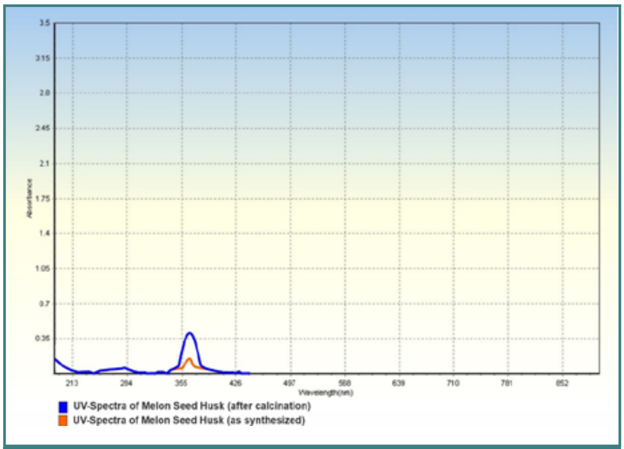

Figure S1. UV-spectra of melon seed husk

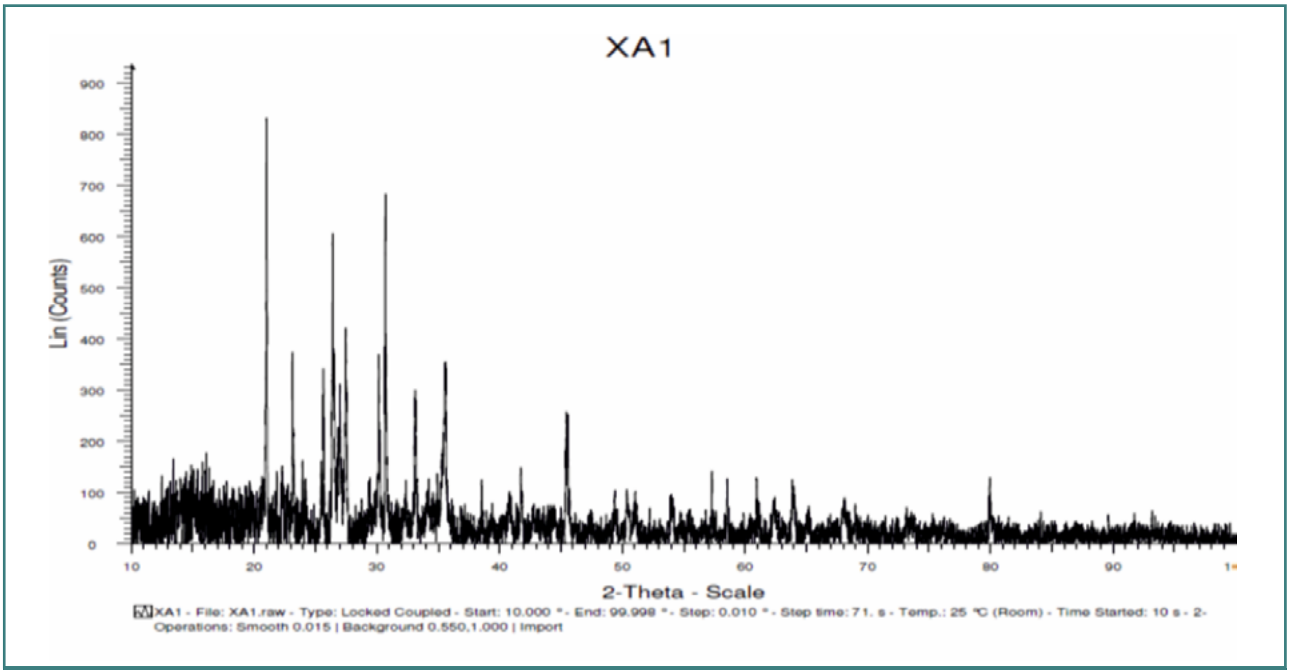

Figure S2. X-RAY diffractogram of melon seed husk-derived silica nanoparticles

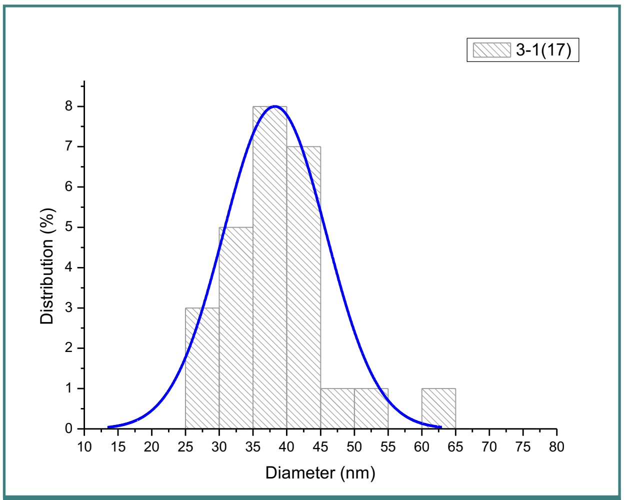

Figure S3. DLS results of melon seed husk-derived silica nanoparticles

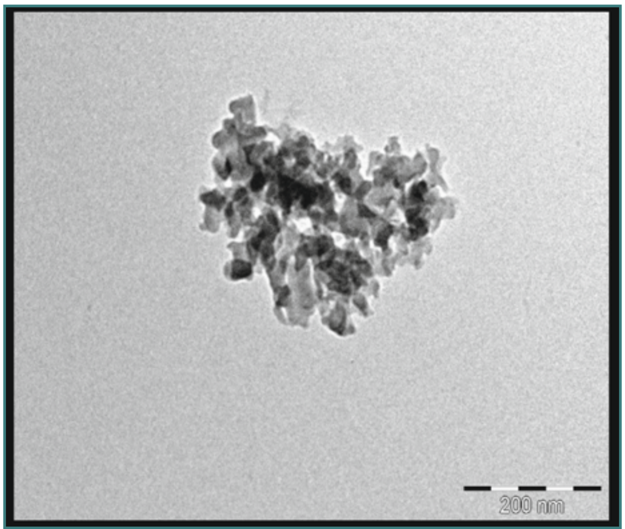

Figure S4. TEM result of melon seed husk-derived silica nanoparticles

© 2024 by the authors. This article is an open access article distributed under the terms and conditions of the Creative Commons Attribution (CC BY 4.0) license.
